# Postdischarge interventions for children hospitalized with severe acute malnutrition: a systematic review and meta-analysis

**DOI:** 10.1093/ajcn/nqaa359

**Published:** 2021-01-30

**Authors:** Christie C A Noble, Jonathan P Sturgeon, Mutsa Bwakura-Dangarembizi, Paul Kelly, Beatrice Amadi, Andrew J Prendergast

**Affiliations:** Blizard Institute, Queen Mary University of London, London, United Kingdom; Zvitambo Institute for Maternal and Child Health Research, Harare, Zimbabwe; Blizard Institute, Queen Mary University of London, London, United Kingdom; Zvitambo Institute for Maternal and Child Health Research, Harare, Zimbabwe; Zvitambo Institute for Maternal and Child Health Research, Harare, Zimbabwe; University of Zimbabwe College of Health Sciences, Harare, Zimbabwe; Blizard Institute, Queen Mary University of London, London, United Kingdom; Tropical Gastroenterology and Nutrition Group, University of Zambia, Lusaka, Zambia; Tropical Gastroenterology and Nutrition Group, University of Zambia, Lusaka, Zambia; Blizard Institute, Queen Mary University of London, London, United Kingdom; Zvitambo Institute for Maternal and Child Health Research, Harare, Zimbabwe

**Keywords:** children, severe acute malnutrition, mortality, discharge, hospitalization, interventions

## Abstract

**Background:**

Children hospitalized with severe acute malnutrition (SAM) have poor long-term outcomes following discharge, with high rates of mortality, morbidity, and impaired neurodevelopment. There is currently minimal guidance on how to support children with SAM following discharge from inpatient treatment.

**Objectives:**

This systematic review and meta-analysis aimed to examine whether postdischarge interventions can improve outcomes in children recovering from complicated SAM.

**Methods:**

Systematic searches of 4 databases were undertaken to identify studies of interventions delivered completely or partially after hospital discharge in children aged 6–59 mo, following inpatient treatment of SAM. The main outcome of interest was mortality. Random-effects meta-analysis was undertaken where ≥2 studies were sufficiently similar in intervention and outcome.

**Results:**

Ten studies fulfilled the inclusion criteria, recruiting 39–1781 participants in 7 countries between 1975 and 2015. Studies evaluated provision of zinc (2 studies), probiotics or synbiotics (2 studies), antibiotics (1 study), pancreatic enzymes (1 study), and psychosocial stimulation (4 studies). Six studies had unclear or high risk of bias in ≥2 domains. Compared with standard care, pancreatic enzyme supplementation reduced inpatient mortality (37.8% compared with 18.6%, *P* < 0.05). In meta-analysis there was some evidence that prebiotics or synbiotics reduced mortality (RR: 0.72; 95% CI: 0.51, 1.00; *P* = 0.049). Psychosocial stimulation reduced mortality in meta-analysis of the 2 trials reporting deaths (RR: 0.36; 95% CI: 0.15, 0.87), and improved neurodevelopmental scores in ≥1 domain in all studies. There was no evidence that zinc reduced mortality in the single study reporting deaths. Antibiotics reduced infectious morbidity but did not reduce mortality.

**Conclusions:**

Several biological and psychosocial interventions show promise in improving outcomes in children following hospitalization for SAM and require further exploration in larger randomized mortality trials. This study was registered with PROSPERO as CRD42018111342 (https://www.crd.york.ac.uk/prospero/display_record.php?RecordID=111342).

## Introduction

Severe acute malnutrition (SAM) is an important cause of childhood mortality and morbidity in low- and middle-income countries in children aged <5 y (1). Children with “complicated” SAM [those with medical complications, an Integrated Management of Childhood Illness (IMCI) danger sign, severe edema, or who fail an appetite test], require inpatient management according to WHO guidelines (2), with discharge to outpatient care when clinical improvement is seen. Children receive ready-to-use therapeutic food (RUTF) at home, with discharge from community programs when nutritional recovery has occurred.

Children leaving hospital following management of complicated SAM have a high ongoing risk of mortality. A systematic review examined outcomes 6–24 mo following discharge from inpatient or outpatient treatment for SAM (3). In the 7 included studies, mortality in children discharged as nutritionally cured was ≤10% during follow-up. Another review reported relapse rates of up to 37%, particularly in the first 6 mo following discharge (4). The Chronic Disease Outcomes after Severe Acute Malnutrition in Malawian Children (ChroSAM) study followed children hospitalized with SAM several years after discharge, and reported persistent growth and functional deficits compared with sibling and community controls (5).

Current interventions following hospital discharge are limited to short-term RUTF, but the existing data highlight the ongoing risk of mortality and relapse in children recovering from SAM. Further interventions during the long window of postdischarge vulnerability could reduce mortality and promote recovery. This systematic review and meta-analysis was undertaken to evaluate the existing evidence for interventions aimed at improving postdischarge outcomes in children treated for complicated SAM.

## Methods

### Search strategy and study selection

PRISMA (Preferred Reporting Items for Systematic Reviews and Meta-Analyses) guidelines were used throughout this review, which followed a prespecified protocol registered with PROSPERO (CRD42018111342). Systematic searches were undertaken using Ovid-MEDLINE, Embase, Global Health, and the Cochrane Central Register of Controlled Trials (CENTRAL) in December 2018. The search strategy was formulated using search terms and subject headings in 4 themes: child or infant, severe acute malnutrition, trial or intervention, and inpatient or discharge (see **Supplemental Methods**). Searches were limited to English language articles published from 1970. The search was repeated in December 2019 to identify new studies. In addition, clinicaltrials.gov was searched to identify any further trials or protocols, with a repeat search in January 2020. Searches of the gray literature used the following websites: No Wasted Lives, WHO, UNICEF, http://www.ennonline.net, and www.acutemalnutrition.org. Five experts were contacted for unpublished data or relevant studies not already identified. Reference lists of shortlisted articles were examined to identify additional studies. If a relevant protocol was identified but no results found, attempts were made to contact the authors for further information.

We included both randomized and nonrandomized trials, and other study types with an appropriate comparison group. Eligible studies included children aged between 6 and 59 mo, hospitalized with SAM, and subsequently discharged. We accepted different definitions of SAM, according to the criteria used by each study at the time. Interventions could be biological or psychosocial but had to be delivered after hospital discharge, even if they were initiated in hospital. Our main outcome of interest was mortality. Studies reporting other clinically relevant outcomes of child health or development were included, whereas those reporting only mechanistic or biochemical outcomes, or indirect outcomes such as maternal depression, were not included.

Studies were excluded if children received nonresidential, community-based outpatient care only (currently recommended only for children with uncomplicated SAM). Studies including children with both complicated and uncomplicated SAM were excluded if the children with complicated SAM comprised <50% or an unknown proportion of the total population, and could not be analyzed separately.

To ensure that we identified approaches that are relevant in the current era, interventions had to be supplemental or distinct from current management, which comprises ambulatory care and RUTF. Studies providing micronutrients in the pre-RUTF era were excluded if the dose was the same or less than is currently provided by RUTF (6) for the majority of children in the study. Studies superseded by current guidelines (e.g., comparing RUTF with local foods, or comparing a prolonged hospital stay with ambulatory rehabilitation) were excluded. Noninferiority studies comparing locally formulated RUTF with standard RUTF were also excluded, because the aim was to identify new interventions of benefit to an individual child, beyond current approaches.

### Data extraction and narrative synthesis

All studies were exported to EndNote for title and abstract screening. Full texts were retrieved and screened for eligibility by 1 reviewer (CCAN), using the inclusion and exclusion criteria specified above. Any uncertainty over the study eligibility was resolved through discussion with a second reviewer (AJP). Data were extracted using a standardized proforma. Risk-of-bias assessments used guidance from the Cochrane Handbook for Systematic Reviews of Interventions (7) and from Cochrane Effective Practice and Organisation of Care (EPOC) resources (8). Risk of bias was assessed at the study level, but where there were differences between the main outcomes, or at different timepoints, these were reported separately.

### Meta-analysis

We prespecified that we would undertake meta-analysis where ≥2 more studies had sufficiently similar interventions and outcomes. We conducted a random-effects meta-analysis to calculate a summary measure of effect (RR), with a 95% CI. Heterogeneity was assessed using Cochran Q test and Higgins *I*^2^ test statistic. Analyses were conducted using Stata Version 16 (StataCorp).

## Results

### Study search

**FIGURE 1:**
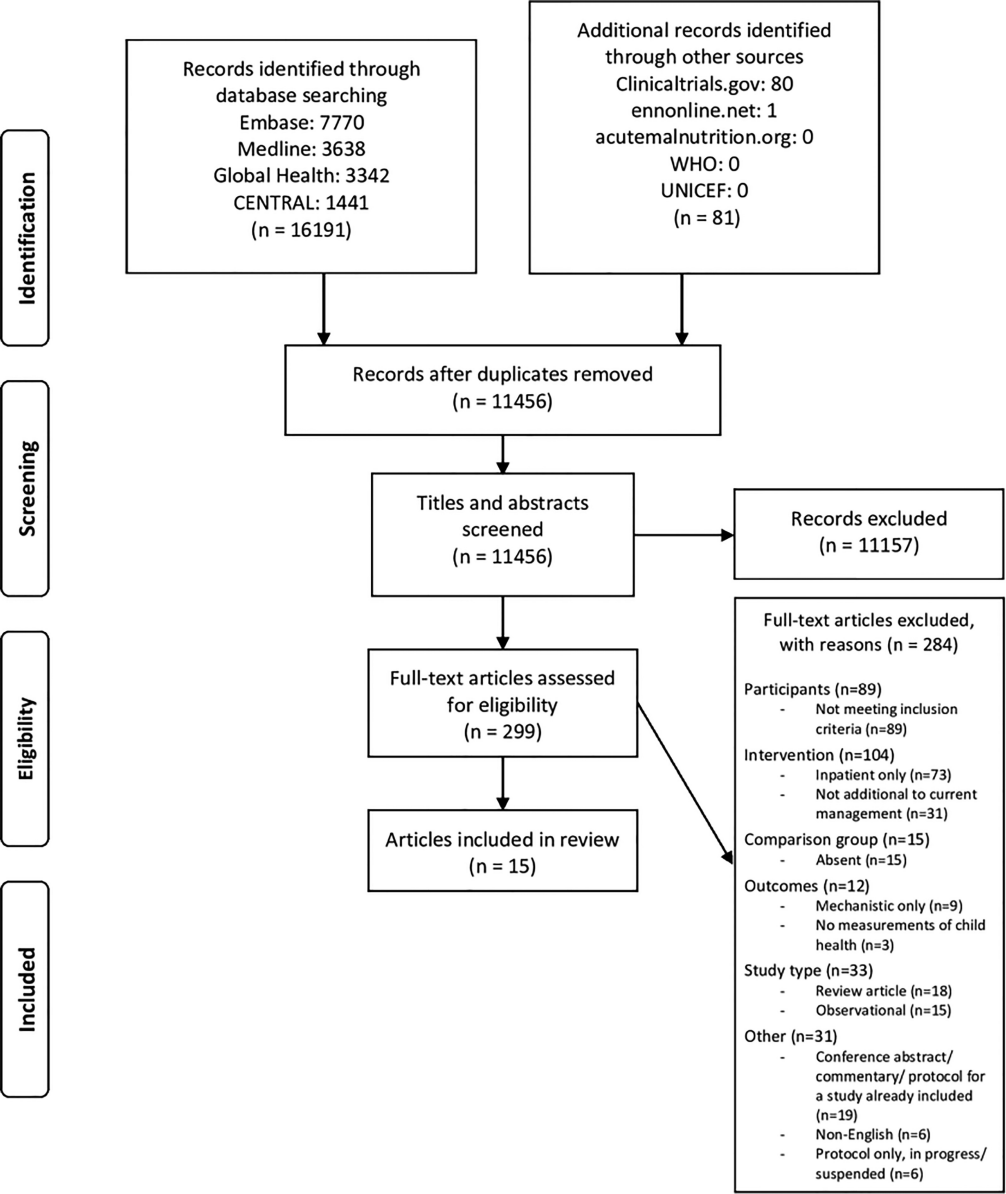
Study selection. PRISMA (Preferred Reporting Items for Systematic Reviews and Meta-Analyses) flow chart of study selection.

Searches identified 11,456 unique records. Of the 299 full-text articles assessed for eligibility, 284 were excluded, mainly because participants did not meet inclusion criteria or interventions were confined to the inpatient period (**Figure 1**). Fifteen articles were included in the review, representing 10 studies in 7 countries, which recruited between 39 and 1781 participants from 1975 to 2015 (**Tables 1** and **2**). Six studies evaluated biomedical interventions: zinc (*n* = 2), probiotics (*n* = 2), antibiotics (*n* = 1), and pancreatic enzymes (*n* = 1). Four studies evaluated psychosocial stimulation.

**TABLE 1:** Details of location, recruitment, inclusion criteria, and definition of SAM in included studies^1^

Article [first author, year, reference(s)]	Country; sites; recruitment dates	Number recruited; age range (median/mean) at recruitment; % HIV positive; % with edema	Growth standards and/or guidance used for admission criteria and/or management	Definition of SAM for purposes of trial	Were only children with complicated SAM admitted?
Grantham-McGregor 1979, 1980, 1983, 1987, 1994 (16–20)	Jamaica; single site; 1975–1977	39; 6–24 (mean 13) mo; unknown; 36%	Wellcome classification	WfA <60%, or <80% with edema	Unclear
Doherty 1998 (21)	Bangladesh; single site; 1995–1996	141; 6–36 (mean 16) mo; unknown; 41%	NCHS growth curves	WfA <60%, or edema	Unclear (all received broad-spectrum antibiotics)
Dutta 2000 (15)	India; single site; 1997–1998	80 (all male); 3–24 (mean 11) mo; unknown; unknown	Harvard Standard	<80% WfA	Unclear (all had diarrhea + dehydration, but other systemic infections excluded)
Kerac 2009 (14)	Malawi; single site; 2006–2007	795; 5–168 (median 22) mo; 46% (5% unknown); 57%	NCHS growth curves, Malawian guidelines 2007, WHO 1999	WfH <70%, or edema, or MUAC <11 cm	No, all children with SAM (50% received parenteral antibiotics)
Nahar 2009 (22)	Bangladesh; single site; 2002–2004	133; 6–24 (mean 12) mo; unknown; 46%	NCHS growth curves, WHO 1999	WfA <50%, or WfH <70%, or edema	Unclear
Hossain 2011 and Nahar 2012 (23, 24)	Bangladesh; single site (4 community clinics); 2005–2007	507; 6–24 (mean 13) mo; unknown; 0% (excluded)	NCHS growth curves, WHO 1999 + 2000, Bangladeshi guidelines 1999	WAZ less than −3	Unclear
Berkley 2016 (25)	Kenya; 4 sites; 2009–2013	1781; 2–59 (median 11) mo; 0%; 17%	WHO 2005	MUAC <11.5 cm (<11.0 cm if <6 mo) or edema	Yes
Bartels 2017 (26)	Malawi; single site; 2014	90; 6–60 (mean 21) mo; 46%; 57%	WHO child growth standards, WHO 2009 + 2013, Malawian guidelines 2014	WHZ less than −3, or MUAC <11.5 cm, or edema	Yes
Grenov 2017 (27)	Uganda; single site; 2014–2015	400; 6–59 (mean 17) mo; 13% (8% unknown); 66%	WHO child growth standards, WHO 2013, Ugandan guidelines 2010	WHZ less than −3, or MUAC <11.5 cm, or edema	Yes
Abessa 2019 (28)	Ethiopia; single site; 2011–2013	339; 6–66 (mean 27) mo; unknown; unknown	NCHS growth curves, Ethiopian guidelines 2007	WfH <70%, or MUAC <11.0 cm if length <65 cm, or edema	Yes

^1^MUAC, midupper arm circumference; NCHS, National Center for Health Statistics median for age; SAM, severe acute malnutrition; WAZ, weight-for-age *z*-score; WfA, weight-for-age; WfH, weight-for-length/weight-for-height; WHO, World Health Organization guidelines; WHZ, weight-for-height *z*-score.

**TABLE 2:** Details of intervention, control group, outcomes, and randomization in included studies^1^

Article [first author, year, reference(s)]	Details of intervention	Control/comparison	Duration of follow-up	Primary outcome(s); main findings	Other outpatient outcomes measured; any significant or key findings	Randomization and blinding
Grantham-McGregor 1979, 1980, 1983, 1987, 1994 (16–20)	Structured play sessions in hospital, 1–2 weekly play sessions and maternal education at home for 3 y	Time-lagged control given standard care	≤14 y	Developmental scores; intervention group had persistently higher scores in most tests (e.g., mean IQ on Stanford–Binet test at 72 mo: 70.9 ± 8.3 vs. 63.8 ± 6.4, *P *< 0.05)	Anthropometry; no differences in anthropometry between groups	Nonrandomized, single-blinded
Doherty 1998 (21)	Zinc 6 mg/kg/d for 30 d, first 15 d given in hospital	Zinc 1.5 mg/kg/d for 15 d then placebo for 15 d, or zinc 6 mg/kg/d for 15 d then placebo for 15 d	90 d	Anthropometry; no difference between groups at 90 d	Mortality, knemometric measures; increased mortality (inpatient and outpatient combined) in the 2 groups receiving 6 mg/kg zinc as inpatients (first 15 d)	Randomized, double-blinded
Dutta 2000 (15)	Zinc 40 mg/d in 3 divided doses, initiated in hospital and continued at home until bottle finished, up to 14 d	Placebo	30 d	Duration and volume of diarrhea, ORS intake; intervention group had reduced duration of diarrhea (70 ± 10 vs. 103 ± 17 h, *P *< 0.05), reduced stool volume, consumed smaller volume of ORS	Anthropometry; small nonsignificant improvements in MUAC and height gain at day 30 (*P* = 0.08 and *P* = 0.06) in intervention group	Randomized, double-blinded
Kerac 2009 (14)	RUTF with “synbiotic” (4 probiotic bacteria and 4 prebiotic fibers) initiated in rehabilitation phase and continued during nutritional rehabilitation	Standard RUTF	∼10 wk	Nutritional cure: WfH >80% of median (NCHS reference) at 2 consecutive visits; no difference between groups (RR: 1.06; 95% CI: 0.93, 1.21; *P* = 0.40)	Mortality, default rate, nutritional failure rate (not cured after 10 wk), weight gain, illness symptoms; trend toward reduced deaths any time after initial admission (RR: 0.71; 95% CI: 0.51, 1.00; *P* = 0.05) and in all outpatient periods (RR: 0.65; 95% CI: 0.42, 1.02; *P* = 0.06) in intervention group	Randomized, double-blinded
Nahar 2009 (22)	Stimulation sessions and maternal education as inpatient, then 18 supervised play sessions as outpatient over 6 mo (mainly home visits)	Time-lagged control given standard care	6 mo	Developmental scores; intervention group had higher scores in mental (mean raw score 103.1 ± 12.1 vs. 94.3 ± 8.8; *P *< 0.05) and psychomotor (mean raw score 67.3 ± 8.1 vs. 63.3 ± 8.2; *P *< 0.05) development using BSID-II at 6-mo follow-up	Anthropometry; higher WAZ at 6 mo in intervention group (*z*-score −3.1 ± 0.9 vs. −3.6 ± 1.2; *P *< 0.05)	Nonrandomized, single-blinded
Hossain 2011 and Nahar 2012 (23, 24)	Community follow-up with stimulation sessions and parental education every 2–4 wk for 6 mo, with or without 3 mo food supplementation (2 groups)	Standard hospital-based or community-based outpatient follow-up, or community-based follow-up with supplementary food for 3 mo (3 groups)	6 mo	Developmental scores, weight gain, follow-up attendance; improved mental development scores (using BSID-II) in 2 stimulation groups combined compared with 3 no-stimulation groups combined at 6 mo	Anthropometry, rehospitalization rate, mortality; improved WAZ score in 2 stimulation groups combined compared with 3 no-stimulation groups combined at 6 mo	Randomized, unblinded (single-blinded on developmental score)
Berkley 2016 (25)	Daily prophylactic co-trimoxazole for 6 mo, initiated during stabilization phase in hospital	Placebo	12 mo	Mortality; no difference between groups (HR: 0.90; 95% CI: 0.71, 1.16; *P* = 0.4)	Illness episodes requiring readmission/outpatient care, clinical syndromes associated with death/illness, culture/malaria testing, adverse effects, anthropometry, Hb; small increase in diarrhea, reduced skin/soft tissue infections, positive urine cultures and confirmed malaria (IRR: 0.60; 95% CI: 0.35, 0.99; *P *< 0.05) in intervention group	Randomised, double-blinded
Bartels 2017 (26)	Pancreatic enzyme replacement therapy 3 times daily prefeeds, initiated in hospital and continued for 28 d	Standard care	28 d	Weight gain; no difference between groups at 28 d	Biochemical markers, mortality; total mortality lower in intervention group (18.6% vs. 37.8%; *P *< 0.05)	Randomised, single-blinded
Grenov 2017 (27)	Sachet of 2 probiotic strains given from hospital admission for 8–12 wk	Placebo	8–12 wk	Number of days of diarrhea during hospitalization; no difference between groups	Incidence, severity and number of days of diarrhea, incidence of pneumonia, weight gain, nutritional recovery, days with fever or vomiting, mortality; fewer days of outpatient diarrhea in intervention group (adjusted effect size −2.2 d; 95% CI: −3.5 to −0.3 d; *P *< 0.05)	Randomised, double-blinded
Abessa 2019 (28)	Stimulation sessions initiated in hospital, then 3 further home visits over 6 mo	Standard care	6 mo	Developmental scores; intervention group had higher fine motor scores on adapted Denver II test at 6 mo (mean score 19.3 ± 3.3 vs. 17.9 ± 4.1; *P *< 0.05)	Anthropometry; no difference between groups at 6 mo	Randomised, single-blinded

^1^BSID-II, Bayley Scales for Infant Development version II; Hb, hemoglobin; IQ, intelligence quotient; IRR, incidence rate ratio; MUAC, midupper arm circumference; NCHS, National Center for Health Statistics median for age; ORS, oral rehydration solution; RUTF, ready-to-use-therapeutic food; WAZ, weight-for-age *z*-score; WfH, weight-for-length/weight-for-height.

### Study characteristics

Five studies were undertaken in sub-Saharan Africa (2 in Malawi, 1 in Kenya, 1 in Ethiopia, and 1 in Uganda), 4 in south Asia (3 in Bangladesh and 1 in India), and 1 in the Caribbean (Jamaica). Only 1 trial recruited children from >1 site. The average age of participants ranged from 11 to 27 mo (mean = 16 mo). Four studies included children outside the target 6–59-mo age range, with the youngest participant at 60 d and the oldest at 168 mo. Of the 4 studies reporting HIV prevalence, 2 reported a prevalence of 46% and 1 of 13%, whereas the other recruited HIV-negative children only. Seven studies reported nutritional edema prevalence of 17% to 66% (mean = 46%); 1 study excluded children with edema.

The definition of SAM varied between studies. Only 2 studies used current WHO criteria (1, 9) and growth standards (10) to define SAM. Three other studies used midupper arm circumference (MUAC): 1 used a MUAC cutoff of 11.5 cm and presence of edema only, whereas 2 studies used a MUAC cutoff of 11 cm, as well as edema and a weight-for-height criterion based on National Center for Health Statistics (NCHS) growth curves (11). The remaining 5 studies recruited children based on weight-for-age [1 using the Wellcome classification (12), 1 using the Harvard Standard, and 3 using the NCHS growth curves]. Two of these studies used weight-for-age only, whereas 3 also recruited children with edema, and 1 also used a weight-for-height criterion.

The 4 most recent studies only admitted children with complicated SAM, as per current WHO guidelines (1, 13). Although not explicit, the other studies likely admitted all children with SAM. It is unclear what proportion of children in these studies would meet current criteria for hospitalization, although there are some indications that allow comparison with current practice. For example, Kerac et al. (14) stated that 50% of admitted children received parenteral antibiotics, and 46% of children were HIV-positive. Dutta et al. (15) included children with watery diarrhea and some dehydration, although excluded children with other systemic infections.

Trials reported primary outcomes of neurodevelopment (*n* = 4), anthropometry (*n* = 3), diarrhea (*n* = 2), and mortality (*n* = 1). A further 7 studies reported mortality; in 5/7 it was prespecified as a secondary outcome. Eight of the 10 studies were randomized; 5 of these were double-blind trials. Four involved single-blind evaluations of all outcomes, and 1 had single-blind evaluation for 1 outcome only. Follow-up periods ranged from 28 d to 14 y. Loss to follow-up was highly variable.

### Risk of bias

Two studies had a low risk of bias across all domains, and 2 studies had an unclear or high risk of bias in 1 domain only (**Table 3**). The other 6 studies, including all 4 studies examining psychosocial interventions, had an unclear or high risk of bias in ≥2 domains.

**TABLE 3:** Risk of bias in included studies^1^

	Random sequence generation	Allocation concealment	Baseline outcome measurements similar	Baseline characteristics similar	Blinding of participants and personnel	Blinding of outcome assessment	Protection against contamination	Incomplete outcome data	Selective reporting	Other risks of bias
Grantham-McGregor et al. 1979, 1980, 1983, 1987, 1994 (16–20)	H	H	L	H	H	L/H 1	U	L/H 4	H	L
Doherty et al. 1998 (21)	L	L	L	L	L	L	L	H	H	L
Dutta et al. 2000 (15)	L	L	L	L	L	L	L	L/H 3	L	L
Kerac et al. 2009 (14)	L	L	L	L	L	L	L	L	L	L
Nahar et al. 2009 (22)	H	H	L	L	H	L	U	H	L	L
Hossain et al. 2011 and Nahar et al. 2012 (23, 24)	L	L	L	L	H	L/H 2	U	H	L	L
Berkley et al. 2016 (25)	L	L	L	L	L	L	L	L	L	L
Bartels et al. 2017 (26)	L	L	U	U	H	L	L	L	L	L
Grenov et al. 2017 (27)	L	L	L	L	L	L	L	U	L	L
Abessa et al. 2019 (13)	L	L	L/U 1	U	H	L	U	H	L	L

^1^L, low risk of bias; H, high risk of bias; U, unclear risk of bias; L/U 1, low/unclear risk of bias; L/H 1, low/high risk of bias. L/H 1, low/high at different timepoints (development only); L/H 2, low for development, high for other outcomes; L/H 3, low for diarrhea, high for anthropometry; L/H 4, low/high at different timepoints; L/U 1, low for primary outcome, unclear for secondary outcome (adjustment in some analyses).

### Study findings

#### Antibiotics

**FIGURE 2:**
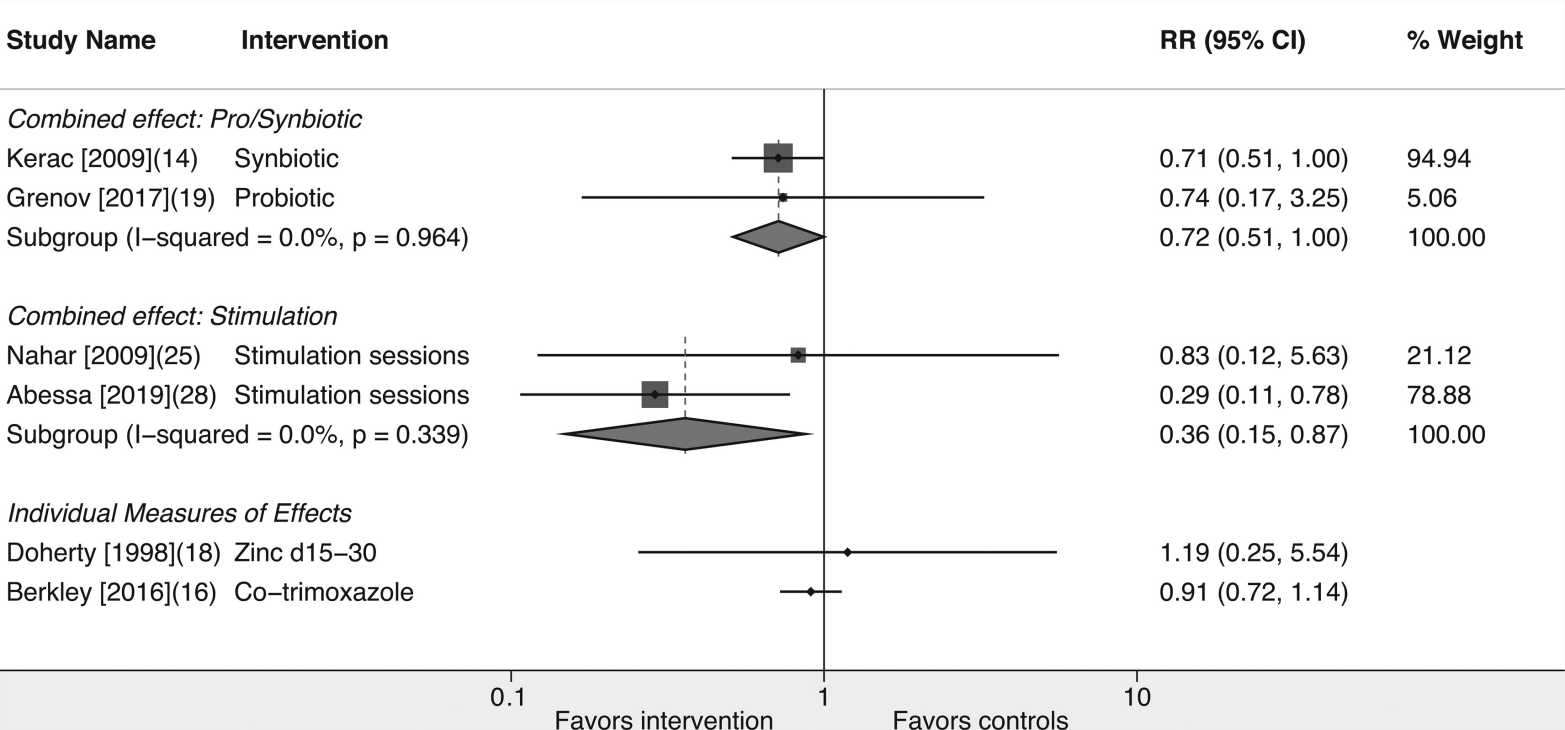
Effects of interventions on postdischarge mortality in children with severe acute malnutrition. Forest plot showing the effects of synbiotics or probiotics, child stimulation, zinc, and co-trimoxazole on mortality after discharge from hospital. Combined effects are from a random-effects meta-analysis.

One study evaluated prophylactic antibiotics. Berkley et al. (25) undertook a placebo-controlled trial of daily co-trimoxazole at 4 sites in Kenya, initiated during stabilization in hospital and continued for 6 mo in HIV-negative children. Co-trimoxazole reduced some infections, including malaria; however, there was no difference in mortality between groups at 12 mo (14% intervention compared with 15% placebo; RR: 0.91; 95% CI: 0.72, 1.14; **Figure 2**).

#### Pancreatic enzymes

Bartels et al. (26) undertook a pilot study of pancreatic enzymes, with children in the intervention group receiving amylase, lipase, and protease 3 times daily for 28 d, compared with standard care. There was no difference in the primary outcome of weight change at 28 d. Mortality (a prespecified secondary outcome) was significantly lower in the intervention group (18.6% compared with 37.8%; *P* < 0.05), although all deaths occurred in the inpatient period. A difference in the proportion of children with edema in intervention and control groups (69% compared with 44%, respectively) might have contributed.

#### Zinc

Two studies evaluated zinc at greater doses than RUTF; at recommended dosing, current RUTF provides 4.7 mg/kg/d zinc (6). Doherty et al. (21) gave 6 mg/kg/d zinc for 30 d (A), with 2 comparison groups receiving either 1.5 mg/kg/d (B) or 6 mg/kg/d (C) zinc for 15 d, followed by placebo in both groups for 15 d. There was no difference in anthropometric measures between groups. Combined inpatient and outpatient mortality was higher in the 2 high-dose zinc groups (4% in B compared with 16% in C compared with 21% in A). Children receiving zinc compared with placebo from days 16 to 30 (group A compared with C) had no evidence of reduced outpatient mortality (RR: 1.19; 95% CI: 0.25, 5.54; Figure 2). Dutta et al. (15) recruited male children with acute watery diarrhea and “some dehydration.” They were provided with either 40 mg/d (average ∼7 mg/kg/d) zinc from a bottle, or placebo, with the bottle continued after discharge for a maximum of 14 d. The intervention group had a significantly shorter duration of inpatient diarrhea (70 compared with 103 h), but there were no significant differences in anthropometry after 30 d. Mortality and outpatient diarrhea were not reported. Meta-analysis of these 2 zinc studies was not undertaken because zinc was provided for different durations during inpatient and outpatient periods, and mortality was only reported in 1 study.

#### Probiotics/synbiotics

Two studies evaluated probiotics or synbiotics. Kerac et al. (14) in Malawi gave 4 probiotics and 4 prebiotics mixed with RUTF, compared with a standard RUTF. There was no difference between groups in nutritional cure, but there was a trend toward reduced mortality in the intervention group following discharge from the index hospitalization (14% compared with 19%; *P* = 0.05). Grenov et al. (27) in Uganda gave 1 sachet of 2 probiotics daily, compared with placebo, for 8–12 wk. There was no difference between groups in the duration of diarrhea during hospitalization (primary outcome), but the intervention group had fewer days with diarrhea during outpatient treatment. Mortality after hospital discharge was similar in the intervention (3/147; 2%) and control groups (4/145; 3%). In meta-analysis, there was evidence that probiotics reduced mortality (RR: 0.72; 95% CI: 0.51, 1.00; *P* = 0.049; Figure 2).

#### Psychosocial interventions

Four studies investigated psychosocial interventions (16–20, 22–24, 28). All studies provided stimulation through child play therapy, together with parental education; interventions were provided at a variable intensity during the outpatient period. Three studies commenced stimulation sessions for the intervention group in hospital (17, 22, 28). Two studies provided home visits (17, 28), whereas 1 provided a mixture of home visits and outpatient clinics (22), and 1 provided outpatient clinics only (23). All control groups received standard care only, with no home visits. The number of outpatient contacts was higher for intervention compared with control groups for 3 studies, and was only similar between groups in the study not providing home visits. All studies reported higher developmental scores in ≥1 domain in the intervention group, and 2 studies also reported higher weight-for-age (22, 23). Two studies reported mortality, which was not a prespecified outcome. In the study by Nahar et al. (22), mortality was similar between the intervention (2/54; 4%) and control group (2/43; 5%). By contrast, in the study by Abessa et al. (28), 5/145 (3%) children in the intervention group died compared with 14/117 (12%) in the nonintervention group (RR: 0.29; 95% CI: 0.11, 0.78). In meta-analysis, there was strong evidence of an effect of psychosocial stimulation on mortality (RR: 0.36; 95% CI: 0.15, 0.87; Figure 2).

## Discussion

Children with complicated SAM have unacceptably high mortality, morbidity, and relapse after discharge from hospital, and long-term risks of impaired neurodevelopment (3–5). SAM has multiple social, environmental, and biomedical determinants and effective long-term management requires a holistic approach. Current WHO guidelines provide current best practice but predominantly focus on inpatient management, and there are limited recommendations for postdischarge convalescence. Approximately 10% of children die during the year after discharge from nutritional care and there is a pressing need for adjunctive interventions to reduce mortality. Our systematic review identified 10 trials conducted over 40 y that evaluated biomedical or psychosocial interventions after hospital discharge. Two biomedical approaches showed some evidence of mortality benefits: pancreatic enzyme supplementation (26), and provision of synbiotics (14). Psychosocial interventions, providing child stimulation and parental education of variable intensity and duration, improved neurodevelopmental scores in all studies (17, 22–24, 28) and significantly reduced mortality in meta-analysis of 2 studies. Collectively these studies indicate several promising biomedical and psychosocial strategies, and the need for additional trials to evaluate novel approaches to improving child survival, health, and development during convalescence.

Pancreatic enzyme supplementation was evaluated in a single-blind pilot study in Malawi (26). Pancreatic insufficiency is common in SAM and is particularly severe in children with edematous malnutrition (29). Lack of digestive enzyme production causes protein and lipid malabsorption, thereby impairing nutritional recovery. There was no difference in the primary outcome of weight gain at 28 d but, unexpectedly, mortality was significantly lower in the intervention group (38% compared with 19%). There was no difference in fecal elastase concentrations between groups, and all deaths occurred in hospital in the first 14 d. It is therefore difficult to explain the mechanism underlying the mortality benefit. One limitation in interpretation is that the proportion of children with edematous malnutrition was significantly higher in the intervention group. It would be valuable to undertake a larger and longer randomized trial of pancreatic enzymes with stratification for edema status.

There is increasing evidence that the gut has a central role in the pathogenesis of SAM, with alterations in intestinal structure, integrity, and microbiome composition likely contributing (30, 31). Optimum interventions to improve gut function and to treat acute and persistent diarrhea are unclear (32). Probiotics might plausibly address enteropathy. There is some evidence for their efficacy in reducing diarrhea in children, although data from low-income countries and malnutrition are lacking (33, 34). Of the 2 probiotic/synbiotic trials identified in our review, one demonstrated ∼2 fewer days of outpatient diarrhea (26% reduction) (27). The other, which gave prebiotics and probiotics formulated into an RUTF, did not reduce diarrhea overall, and severe inpatient diarrhea was increased (14). There was no evidence of probiotic-related sepsis in either trial. In meta-analysis there was some evidence that probiotics reduced mortality, driven by lower postdischarge mortality in Malawi. These findings suggest that probiotics show promise, but greater clarity is needed on which species, dose, and duration to use, particularly in low-income settings. Zinc is also a plausible gut-focused intervention, evaluated in 2 trials. One trial reported that zinc reduced diarrhea in the inpatient period (15), but neither measured diarrhea after discharge. The other trial (21) reported more deaths in the 2 groups receiving higher-dose zinc in the first 15 d, with most appearing to be inpatient deaths from sepsis. Higher-dose zinc had no mortality benefit compared with placebo when continued after hospital discharge. The authors hypothesized that zinc could have harmful effects on the immune response during sepsis and could impede absorption of other micronutrients. The dose of 6 mg/kg/d is higher than current provision in RUTF (∼4.7 mg/kg/d) (6), and was likely started earlier in treatment. Further understanding of the role of zinc in SAM recovery is needed.

The largest trial investigated whether prophylactic co-trimoxazole reduces mortality, because infections are the main cause of death during recovery from SAM (30). Co-trimoxazole was chosen because of its reported benefits in HIV-infected children (35–38), and its immunomodulatory properties (39, 40). However, co-trimoxazole prophylaxis for 6 mo postdischarge did not improve mortality or growth outcomes in HIV-negative children with SAM, although there was a lower incidence of malaria and some bacterial infections (25). There is mixed evidence for the benefit of short antibiotic courses in uncomplicated severe acute malnutrition, with reduced mortality and increased nutritional recovery seen in Malawi (41) but not in Niger, where the rates of HIV and edematous malnutrition were much lower (42). Children discharged from hospital can be colonized or infected with bacteria that are resistant to commonly used antibiotics, and a different antimicrobial strategy could be required to reduce severe bacterial infections, and other infections including tuberculosis.

Global attention is increasingly moving beyond child survival, to ensuring that children thrive. Sustainable Development Goal 4 (43) now includes a target for quality early childhood development. There is evidence of a sustained detrimental impact of SAM on developmental potential and long-term cognitive function, which is exacerbated by psychosocial deprivation (44, 45). Child play is included in the WHO guidelines for hospital management of SAM, with advice that the caregiver continue the intervention after discharge (2). Two randomized and 2 nonrandomized trials examined the impact of delivering stimulation sessions through child play together with parental education, either at home or in clinics; all showed an improvement in ≥1 neurodevelopmental domain. It is challenging to conduct high-quality, long-term studies in SAM, and all 4 studies had a high risk of bias in ≥2 domains, with substantial loss to follow-up and an unclear risk of contamination. The interventions were of very variable intensity (from 3 visits over 6 mo, to 1–2-weekly sessions over 3 y), making comparability difficult. Of the 2 trials reporting mortality, 1 showed significantly lower mortality in the intervention group compared with the control group. Mortality was not a prespecified outcome in this randomized trial, and the mechanism underlying mortality reduction is uncertain, given that there was no difference in anthropometry between groups (primary outcome). Whether a strategy of home visits in itself reduces mortality, regardless of the content of the visits, needs to be investigated, because the control group received standard care without home visits. Collectively, it is evident from this systematic review that psychosocial interventions improve long-term outcomes in SAM, but the optimum nature, timing, and intensity of intervention is unclear.

Several other relevant trials did not meet inclusion criteria for our review. A cluster-randomized trial in the Democratic Republic of Congo found that monthly cash transfers significantly improved nutritional outcomes and reduced relapse at 6 mo (46), due to improved dietary diversity and food security. Water treatment packages implemented alongside outpatient therapeutic feeding programs improved recovery from uncomplicated SAM in Pakistan and Chad (47, 48). The causal pathway is unclear, because there were no significant reductions in diarrhea. Preliminary studies have adjusted RUTF composition to improve ω-6 and ω-3 fatty acid balance (49–51), and given mesalazine to treat enteropathy (52). Consideration has also been given to the optimum timing of antiretroviral therapy initiation in HIV-infected children during nutritional rehabilitation (53–56). Finally, a package of health and nutrition interventions, including lipid-based nutritional supplements, malaria chemoprophylaxis, deworming, and zinc, given to children after completing moderate acute malnutrition treatment, did not improve outcomes at 1 y (57).

We sought to find interventions that were supplemental to current SAM management, so that they would have relevance to the current era. The SAM treatment “landscape” has evolved over recent years, with the introduction of RUTF and community-based management of acute malnutrition, and there is a large body of research that has been superseded. For example, we did not include studies that compared inpatient and home-based rehabilitation, because home-based care after initial stabilization has been established to be cost-effective and equally efficacious in promoting nutritional recovery (58, 59). We did not discuss studies comparing different forms of food supplementation during home-based care, because provision of RUTF has been established as best practice in WHO guidance (60–62). In addition, there has recently been a focus on using locally produced RUTF with alternative ingredients, to increase capacity and reduce program costs (63–68). These topics were outside the scope of this review.

Several biomedical and psychosocial approaches appear promising and require further exploration in larger randomized trials designed to assess mortality. SAM is a complex condition with both medical and social components, and it is likely that a holistic package of care is needed to address its multiple determinants. We did not identify any studies that combined both biomedical and psychosocial approaches. An enabling environment is likely needed to promote effective recovery from SAM, with interventions that address the root causes of poverty, food insecurity, poor maternal physical and mental health, and gendered household dynamics. Integrated care is a cornerstone of child health, not just in SAM, as typified by the IMCI strategy (69) and more recent Nurturing Care Framework (70). As such, we would advocate for future convalescent packages of care to be tested.

A key limitation of this review was the variation in definitions of complicated SAM between studies. In addition, inpatient and outpatient management was not always consistent with current practice. Prior to availability of outpatient RUTF, children would receive supervised milk feeds in hospital for longer durations than currently, and would generally be discharged when they reached a target weight, in contrast to current practice. We felt it was important to include as many relevant studies as possible, but there is likely an impact on the comparability of the studies and their applicability to the current era. Another limitation is that the interventions were of variable intensity and duration, with some initiated early during hospitalization. Some key primary outcomes were measured solely during the inpatient period, or after very short follow-up periods. It was therefore difficult to form conclusions about whether some interventions had longer-term benefits. Over half the studies had a high or unclear risk of bias, and trials varied in size, location, SAM definition, HIV prevalence, primary outcome, and duration of follow-up, meaning the findings of the review need to be interpreted with caution.

In summary, there is currently limited evidence to inform convalescence in children with complicated SAM following discharge from hospital. Only 10 trials from the past 5 decades met our inclusion criteria, despite the urgent need for new approaches to reduce high postdischarge mortality. Several biomedical and psychosocial approaches show promise, but further exploration is required. It seems likely that a package of interventions, including both biomedical and psychosocial elements, would bring most benefit, in view of the multifaceted factors underlying mortality following discharge from hospital in this vulnerable population.

## Supplementary Material

nqaa359_Supplemental_File

## Data Availability

Data described in the manuscript, code book, and analytic code will be made available upon request.

## Notes

JPS and AJP are funded by the Wellcome Trust (grants 220566/Z/20/Z and 108065/Z/15/Z).

Supplemental Methods are available from the “Supplementary data” link in the online posting of the article and from the same link in the online table of contents at https://academic.oup.com/ajcn/.

Abbreviations used: IMCI, Integrated Management of Childhood Illness; MUAC, midupper arm circumference; NCHS, National Center for Health Statistics; RUTF, ready-to-use therapeutic food; SAM, severe acute malnutrition.
