# Supplementary material for: Postdischarge interventions for children hospitalized with severe acute malnutrition: a systematic review and meta-analysis
Source: Am J Clin Nutr. 2021 Jan 30;113(3):574–85. doi: 10.1093/ajcn/nqaa359 (PMC7948836; doi:10.1093/ajcn/nqaa359)
Supplement: nqaa359_Supplemental_File [file nqaa359_supplemental_file.docx]

**Online Supplementary Material**

**Supplementary Methods**

The following search strategy was used in OVID – Medline:

1. child* OR Child, preschool/
2. paediatric*
3. pediatric*
4. infant* OR Infant/
5. severe* adj3 (maln* OR wast*)
6. acute* adj2 maln*
7. protein-energy malnutrition OR Protein energy malnutrition/
8. protein-calori* malnutrition
9. malnutrition OR Malnutrition/
10. malnourish* OR mal-nourish*
11. undernourish* OR under-nourish*
12. undernutrition OR under-nutrition
13. kwashiorkor OR Kwashiorkor/
14. marasmus OR marasmic
15. wasted OR wasting
16. (nutrition* adj2 oedema*)
17. (nutrition* adj2 edema*)
18. Severe acute malnutrition/
19. Wasting syndrome/
20. Intervention*
21. Trial*
22. Treat*
23. Therap*
24. Inpatient* OR In-patient*
25. Outpatient* OR Out-patient*
26. Communit*
27. Home*
28. Hospital*
29. Therapeutic feeding cent*
30. Therapeutic feeding program*
31. Discharge*
32. Rehabilitat*
33. Stabili?ation
34. Recover*
35. Aftercare OR after-care
36. 1 OR 2 OR 3 OR 4
37. 5 OR 6 OR 7 OR 8 OR 9 OR 10 OR 11 OR 12 OR 13 OR 14 OR 15 OR 16 OR 17 OR 18 OR 19
38. 20 OR 21 OR 22 OR 23
39. 24 OR 25 OR 26 OR 27 OR 28 OR 29 OR 30 OR 31 OR 32 OR 33 OR 34 OR 35
40. 36 AND 37 AND 38 AND 39

Limit 40 to… (English language and humans and yr=”1970-Current” and (“all infant (birth to 23 months)” or “all child (0 to 18 years)”))
